# Supplementary material for: Longitudinal single-cell analysis of a myeloma mouse model identifies subclonal molecular programs associated with progression
Source: Nat Commun. 2021 Nov 3;12:6322. doi: 10.1038/s41467-021-26598-w (PMC8566524; doi:10.1038/s41467-021-26598-w)
Supplement: Supplementary file 1 — SupplementaryInformation [file 41467_2021_26598_MOESM1_ESM.pdf]

Supplementary Information

Longitudinal single-cell analysis of a myeloma mouse model identifies subclonal molecular programs associated with progression

Danielle C. Croucher[1,2], Laura M. Richards[1,2,6], Serges P. Tsofack[1,6], Daniel Waller[3], Zhihua Li[1], Ellen Nong Wei[1], Xian Fang Huang[3], Marta Chesi[4], P. Leif Bergsagel[4], Michael Sebag[3], Trevor J. Pugh[1,2,5,7*], Suzanne Trudel[1,2,7*]

[1]Princess Margaret Cancer Centre, University Health Network, Toronto, ON, Canada. [2]Department of Medical Biophysics, University of Toronto, Toronto, ON, Canada. [3]Department of Medicine, McGill University, Montréal, QC, Canada. [4]Division of Hematology/Oncology, Mayo Clinic, Scottsdale, AZ, USA. [5]Ontario Institute for Cancer Research, Toronto, ON, Canada. [6]These authors contributed equally to this work: Laura M. Richards, Serges P. Tsofack, [7]These authors jointly supervised this work: Trevor J. Pugh, Suzanne Trudel

Contents:

- Supplementary Table 1
- Supplementary Figures 1-8

Supplementary Table 1: DNA oligonucleotides used in this study for GCN2 CRISPR knockouts.

Clone	Target Sequence	Genomic Location	Primers
GCN2-A	CTATGACAGCGACATACTGAAGG	chr15:40001203 (exon 21)	5’ CACCGCTATGACAGCGACATACTGA 3’<br>5’ AAACTCAGTATGTCGCTGTCATAGC 3’
GCN2-B	GAACTGGCCAAGAAACACTGTGG	chr15:39943459 (exon 3)	5’ CACCGAACTGGCCAAGAAACACTGT 3’<br>5’ AAACACAGTGTTTCTTGGCCAGTTC 3’

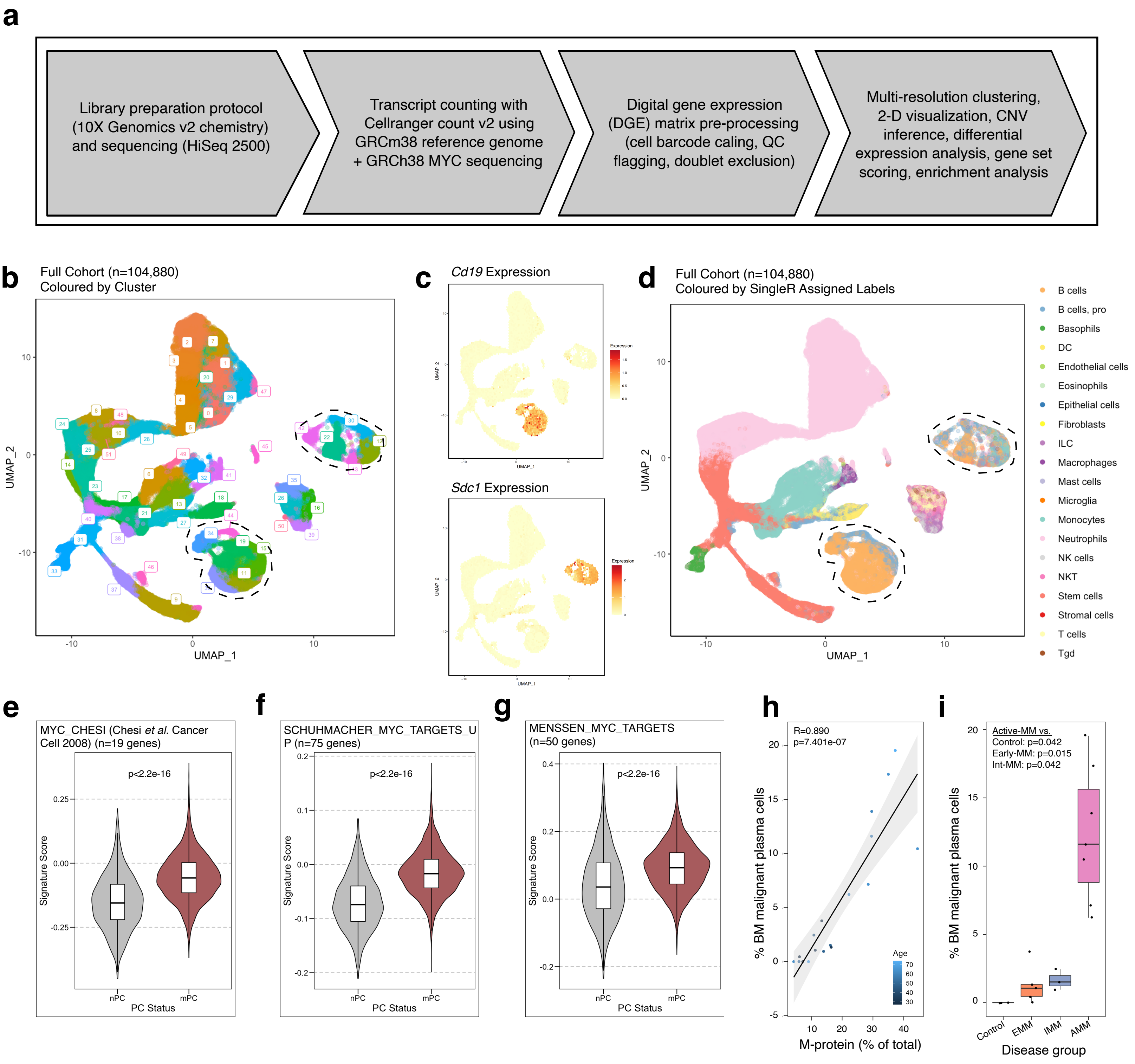

Supplementary Figure 1: Study design and disease stage classifications of Vκ*MYC mice. (a) Summary of computational workflow used for scRNA-seq analysis. **(b)** UMAP visualization of 104,880 bone marrow cells from 18 samples coloured by transcriptional cluster. Suspected plasma cell and B cell clusters are circled. **(c)** Log-normalized expression of select plasma cell (*Sdc1*) and B cell (*Cd19*) marker genes. **(d)** UMAP visualization of 104,880 bone marrow cells from 18 samples coloured by annotated cell types as predicted using SingleR analysis. Suspected plasma cell and B cell clusters are circled. **(e-g)** MYC-specific gene signature scores (Chesi *et al*. Schuhmacher *et al*. and Menssen *et al*.) for plasma cells calculated using Seurat's AddModuleScore comparing normal plasma cells (nPC) vs. malignant plasma cell (mPC) populations. Statistical comparisons of scores were performed using a Kruskal-Wallis rank sum test. **(h)** Pearson correlation (cor.test, two-sided) between the proportion of malignant plasma cells in the bone marrow (BM) or each mouse and serum M-protein measurement. The linear regression line is plotted in black with confidence interval shaded grey. Each dot represents one mouse and is coloured by age. **(i)** Disease burden across cohort as determined by proportion of malignant plasma cells in the BM. Statistical comparison of multiple groups were performed using a Wilcoxon rank sum test (two-sided) corrected for multiple testing (Benjamini-Hochberg). Only P values for statistically significant

comparisons are listed. Data points represent measurements from biologically-independent animals (Control (n=3), early-MM (n=5), int-MM (n=3), and active-MM (n=7)). Boxplots in (e), (f), (g), and (i) represent the distribution of each measurement within defined groups, where the central rectangle spans the interquartile range, the central line represents the median, and “whiskers” above and below the box show the value 1.5x the interquartile range. Source data are provided in SourceData_FigS1.xlsx. EMM: early-MM, IMM: intermediate-MM, AMM: active-MM.

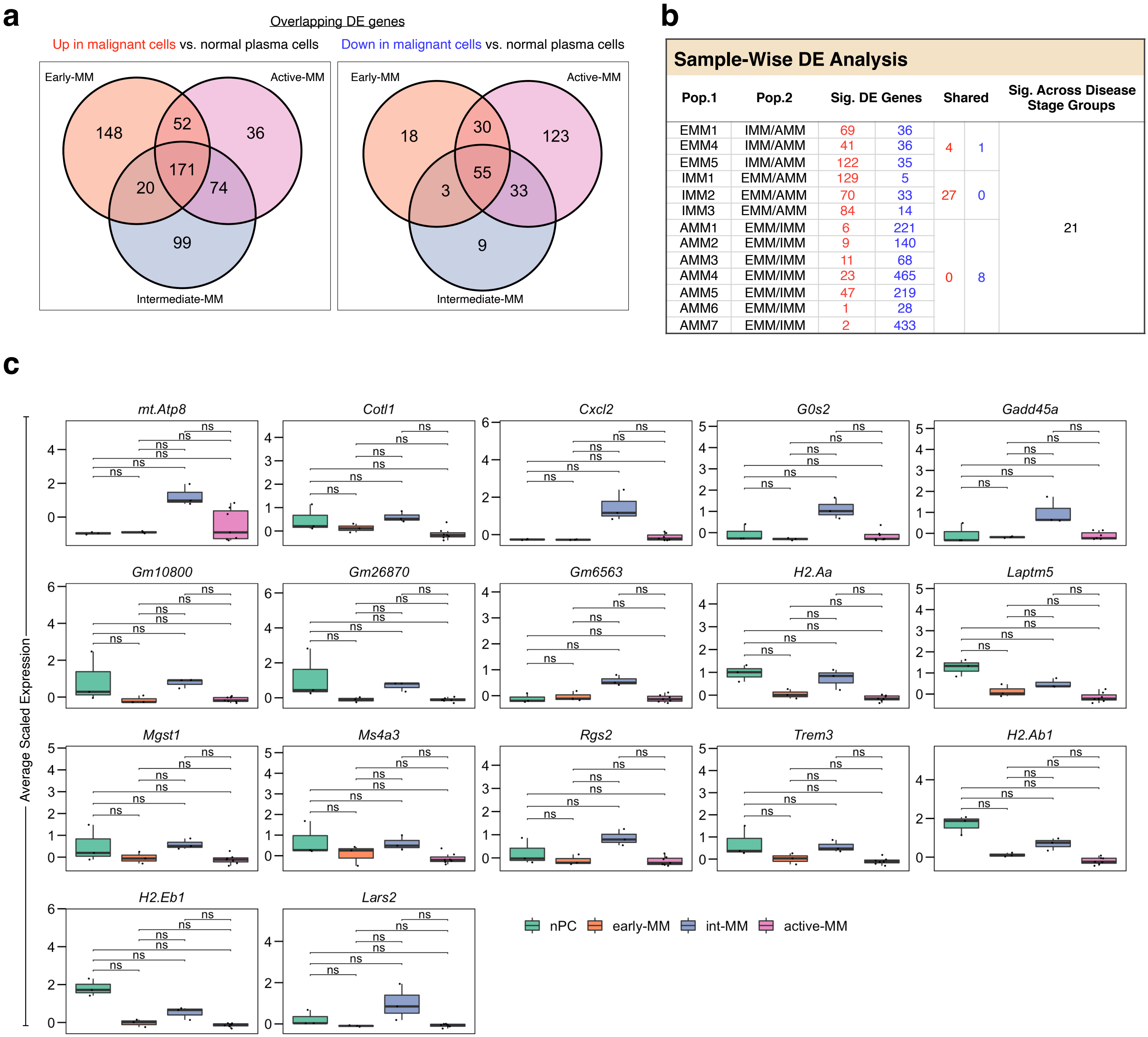

Supplementary Figure 2: Transcriptional signatures of malignant cells from Vκ*MYC mice. (a) Venn diagram depicting overlaps from differential expression (DE) analysis comparing disease stage-specific malignant cells to normal plasma cells (FDR<0.05). **(b)** Analysis strategy and results used to define disease stage-specific differentially expressed genes. Each row shows the two malignant cell populations compared in the DE analysis ("Pop.1", "Pop.2") and the corresponding results ("Sig. DE Genes", red=up, blue=down). Differentially expressed genes shared by all samples within a given disease stage group are shown ("Shared", red=up, blue=down) along with the results of a statistical comparisons between disease stage groups ("Sig. Across Disease Stage Groups", FDR<0.05). Note, EMM3 only contained 4 malignant cells and thus was not included in sample-specific DE analysis and was omitted from all downstream analyses. **(c)** Expression of differentially expressed genes overlapping within disease stage-specific samples, but not statistically significant when compared across disease stage groups. Statistical comparisons were performed using a two-sided t-test with subsequent correction for multiple testing (Bonferroni, ns=not significant). Black data points represent mean expression of respective genes in cells from each biologically-independent animal (Cont1=44 cells, Cont2=72 cells, Cont3=148 cells, EMM1=45 cells, EMM4=52 cells, EMM5=71 cells, IMM1=206 cells, IMM2=88 cells, IMM3=149 cells, AMM1=2,003 cells, AMM2=830 cells, AMM3=1,379 cells, AMM4=822 cells, AMM5=302 cells, AMM6=323 cells, AMM7=310 cells). Boxplots in (c) represent the distribution of each measurement within defined groups, where the

central rectangle spans the interquartile range, the central line represents the median, and “whiskers” above and below the box show the value 1.5x the interquartile range. Source data are provided in SourceData_FigS2.xlsx.

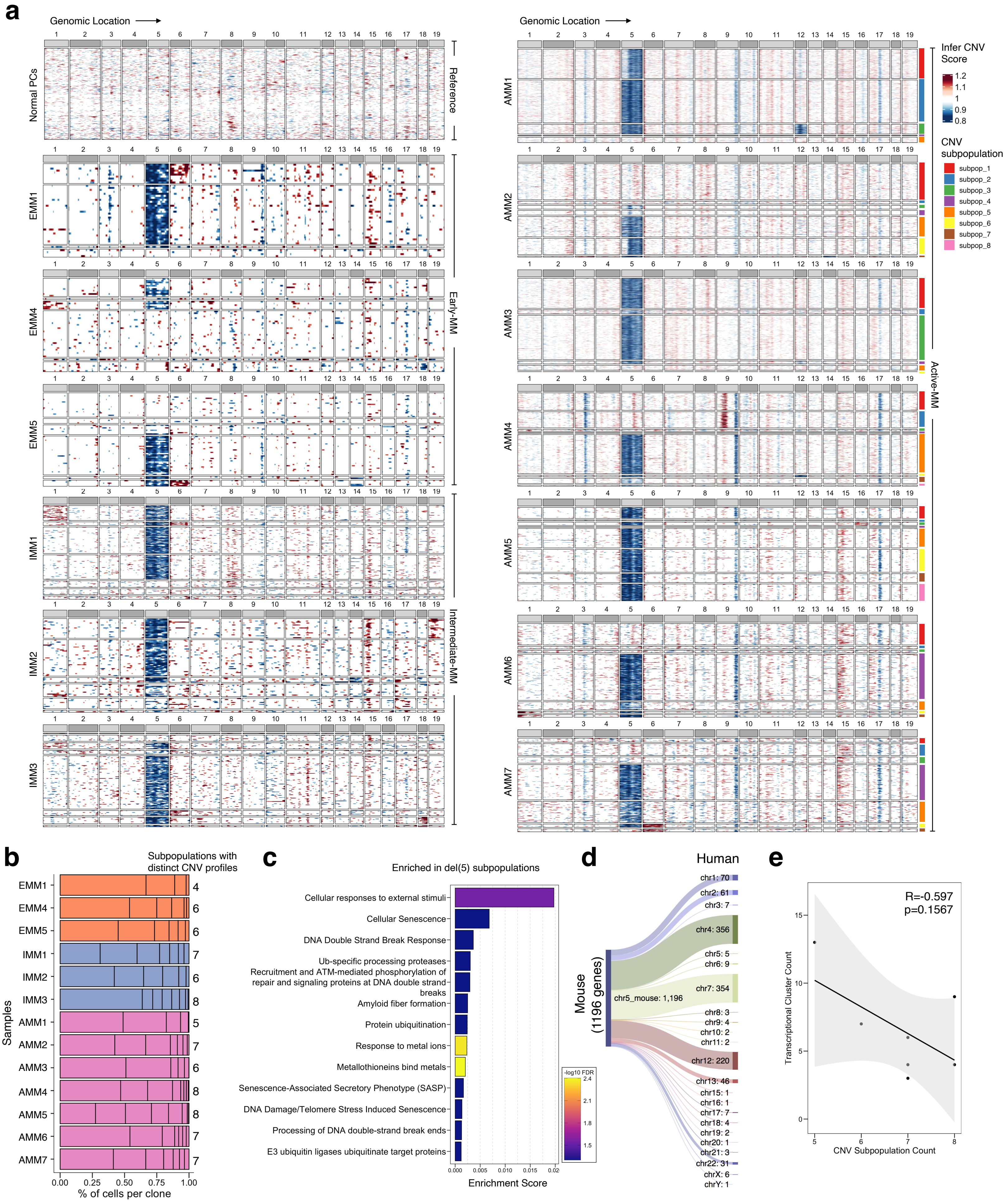

Supplementary Figure 3: Inferred CNV analysis of scRNA-seq data from Vκ*MYC mouse tumours. (a) Heatmap of genome-wide copy number variation inferred from scRNA-seq data of malignant plasma cells as determined using InferCNV. Malignant cells from each sample are plotted in individual heatmaps, where rows represent the inferred CNV

profile of each individual cell, grouped by CNV subpopulation. Vertical colours depicted along right side of heatmaps for active-MM samples correspond to CNV-driven subpopulation colours in Figure 3c-d. Inferred CNV results for normal plasma cells, which were used as reference, are depicted in the top left heatmap. **(b)** Bar plot showing the CNV-level subpopulation composition of each sample (subpopulation counts are listed beside each bar). **(c)** Enriched terms from Reactome analysis (FDR<0.05) using genes upregulated in cells with deletion of chromosome 5. **(d)** Sankey plot depicting distribution of mouse chromosome 5 genes across the human genome. Mouse-human orthologues were determined using Ensembl BioMart (GRCm38.p6 to GRCh38.p13). **(e)** Pearson correlation (cor.test, two-sided) between the number of CNV subpopulations (x-axis) and the number of transcriptional clusters (y-axis). The linear regression line is plotted in black with confidence interval shaded grey. Each dot represents one active-MM mouse. Source data are provided in SourceData_FigS3.zip. EMM: early-MM, IMM: intermediate-MM, AMM: active-MM.

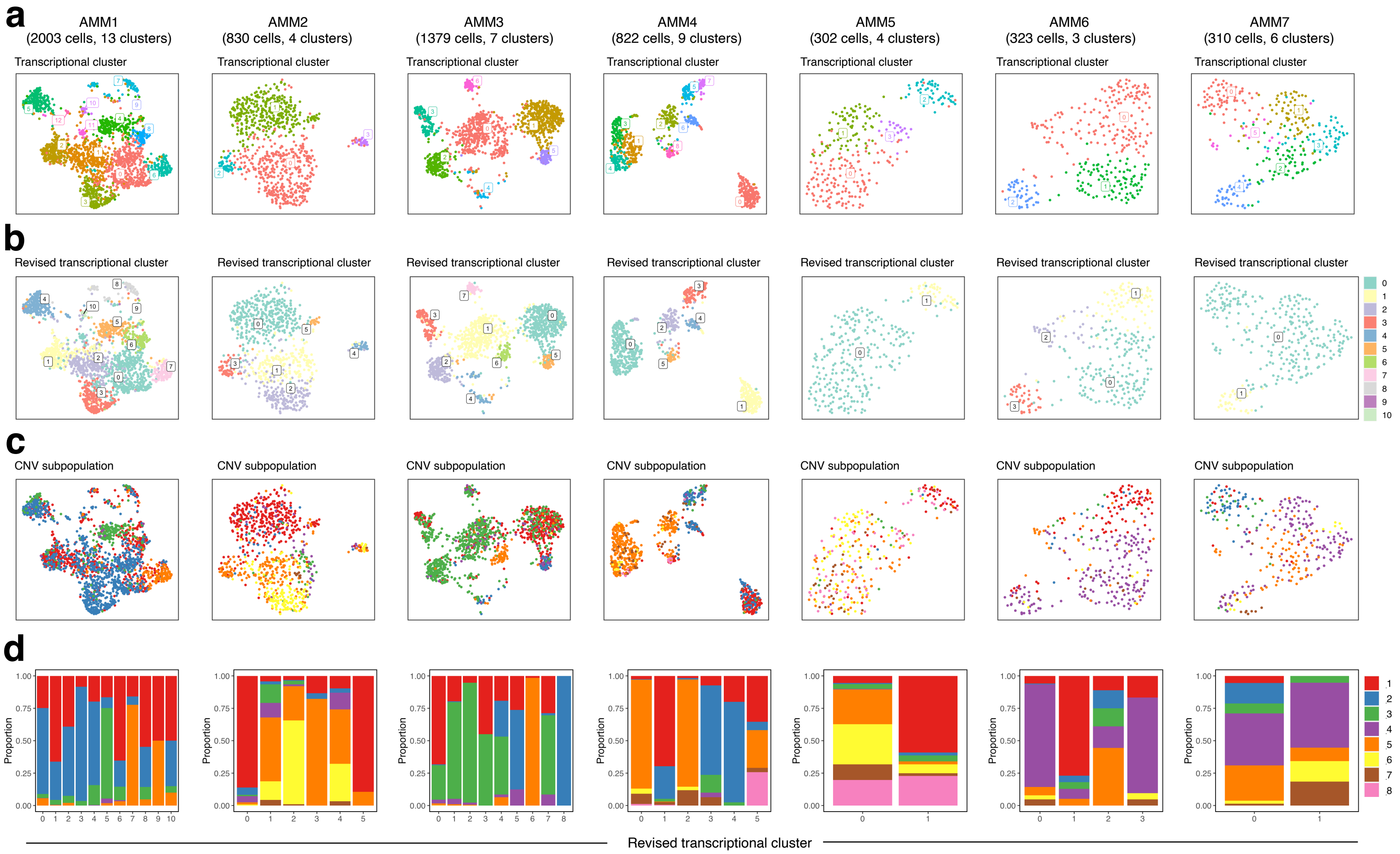

Supplementary Figure 4: Impact of removing dissociation, mitochondrial, ribosomal and cell cycle genes on transcriptional heterogeneity. (a) UMAP visualization of malignant cells from each active-MM mouse coloured by original transcriptional cluster from Fig. 3b. **(b)** UMAP visualization of malignant cells from each active-MM mouse coloured by revised transcriptional clusters upon removal of dissociation, mitochondrial, ribosomal, and cell cycle genes. After removal of these genes, data were re-analyzed using a multi-resolution clustering approach as described in Methods. The resulting clusters were then mapped back to UMAP plots generated from (a) for comparison. **(c)** UMAP visualization of malignant cells from each active-MM mouse coloured by CNV subpopulation. **(d)** Bar plot showing the distribution of CNV subpopulations (fill) across revised transcriptional clusters (x-axis). Results are organized for each active-MM mouse in columns, with subject names and number of cells/transcriptional clusters listed above. Source data are provided in SourceData_FigS4.xlsx. AMM: active-MM.

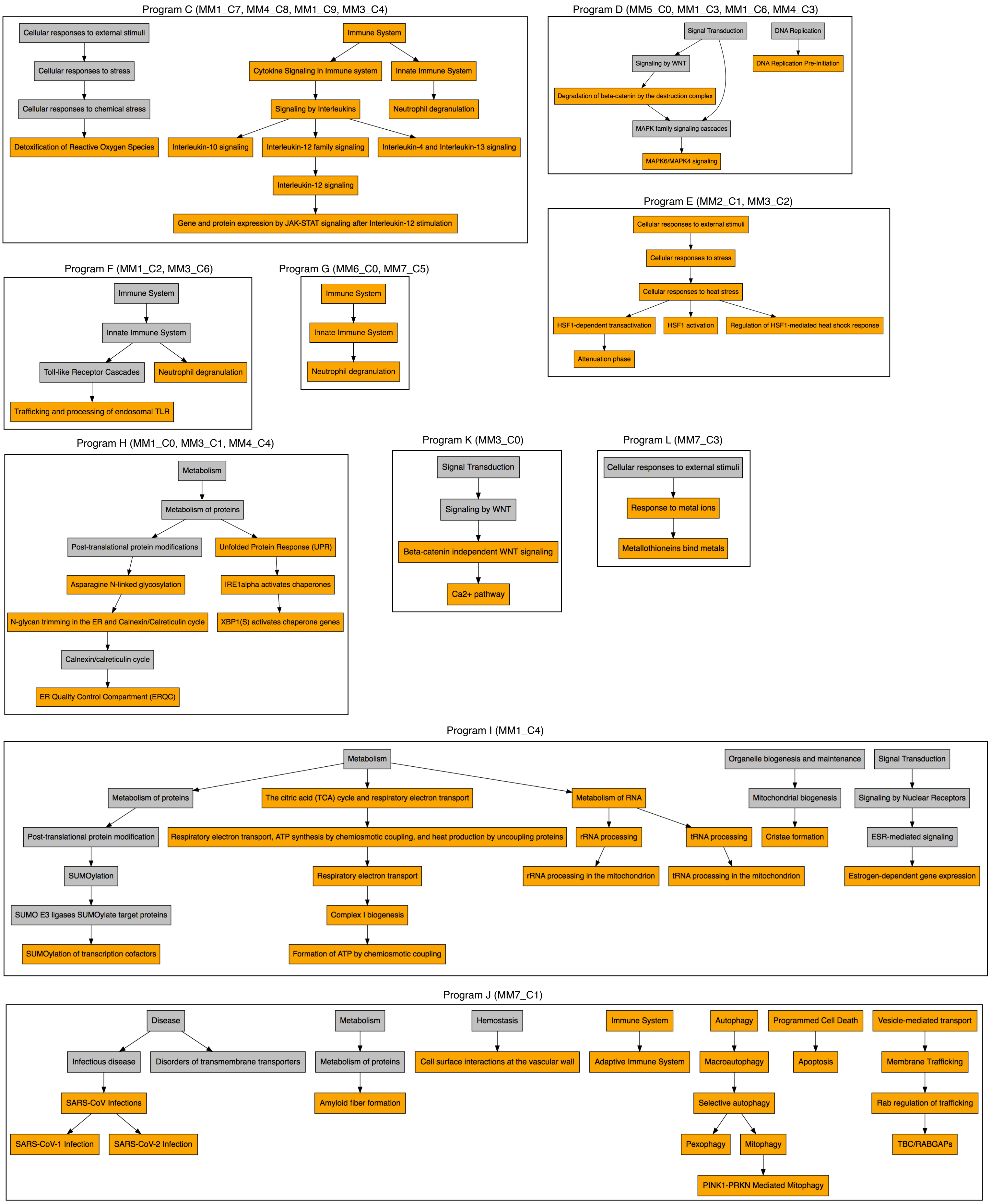

Supplementary Figure 5: Subclonal transcriptional programs in Vκ*MYC mice with active-MM mice. Map of Reactome terms with significant enrichment in malignant cell clusters from Divergent programs defined in Fig. 4a (associated clusters are listed next to Program name). The full hierarchy of each Reactome pathway is shown for context but only significantly enriched shared pathways are highlighted in orange.

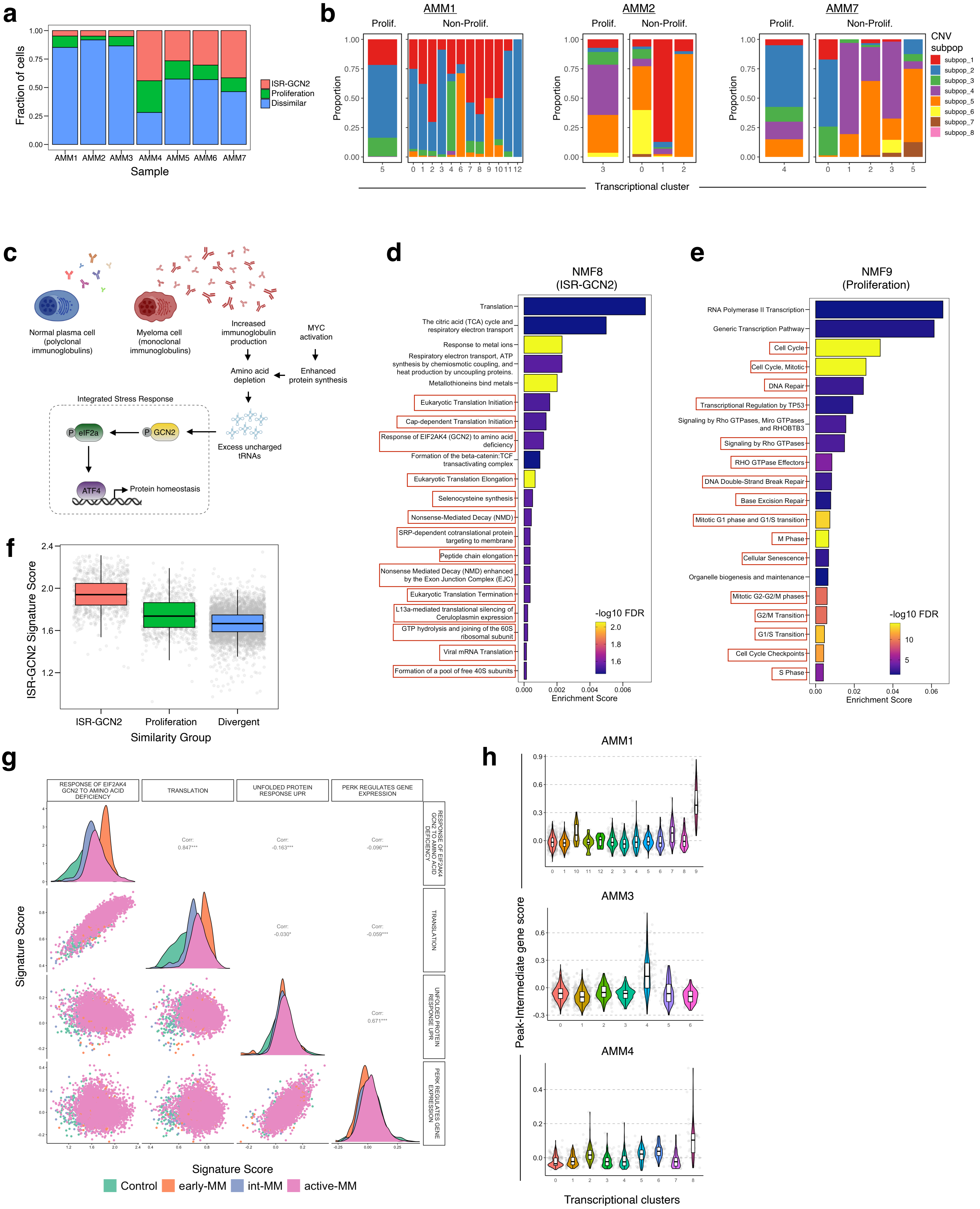

Supplementary Figure 6: Relationship between transcriptional clusters in Vκ*MYC mice with active-MM. (a) Bar plot showing the distribution of Similarity Programs for each Vκ*MYC mouse with active-MM. **(b)** Bar plot showing the

distribution of CNV subpopulations (fill) across transcriptional clusters (x-axis) separated into two plots for proliferative clusters vs. non-proliferative clusters. Results are organized for active-MM mouse AMM1, AMM2, and AMM7 in columns. **(c)** Schematic of proposed role of ISR-GCN2 in Vκ*MYC disease progression. Graphics created in part using BioRender.com. **(d-e)** Reactome analysis of top 50 features from non-negative matrix factorization (NMF) signatures NMF8 (d) and NMF9 (e), which respectively correspond with Similarity Program A (ISR-GCN2) and Similarity Program B (Proliferation). Red boxes indicated overlapping Reactome terms between the results of similarity analysis in Fig. 4a and NMF analysis. **(f)** Gene signature scoring for ISR-GCN2 gene set calculated using Seurat's AddModuleScore across Similarity Groups (ISR-GNC2=894 cells, Proliferation=718 cells, Divergent=4,357 cells). **(g)** Pairwise-correlations between listed gene signature scores computed for each cell using Seurat's AddModuleScore. The corresponding Pearson correlations are listed with significance (*P<0.05, ***P<0.001). **(h)** Gene signature scoring across transcriptional clusters for "Peak-Intermediate" gene set developed from analysis in Fig. 2e. Scores were calculated for each cell using Seurat's AddModuleScore. Results are presented for AMM1, AMM3 and AMM4 as box plots within violin plots to depict the distribution of scores within each cluster. Clusters with the highest "Peak-Intermediate" scores (AMM1_C9, AMM1_C7, AMM3_C4, AMM4_C8) correspond to clusters in Divergent Program C from Fig. 4a. Boxplots in (f) and (h) represent the distribution of each measurement within defined groups, where the central rectangle spans the interquartile range, the central line represents the median, and "whiskers" above and below the box show the value 1.5x the interquartile range. Source data are provided in SourceData_FigS6.xlsx. AMM: active-MM.

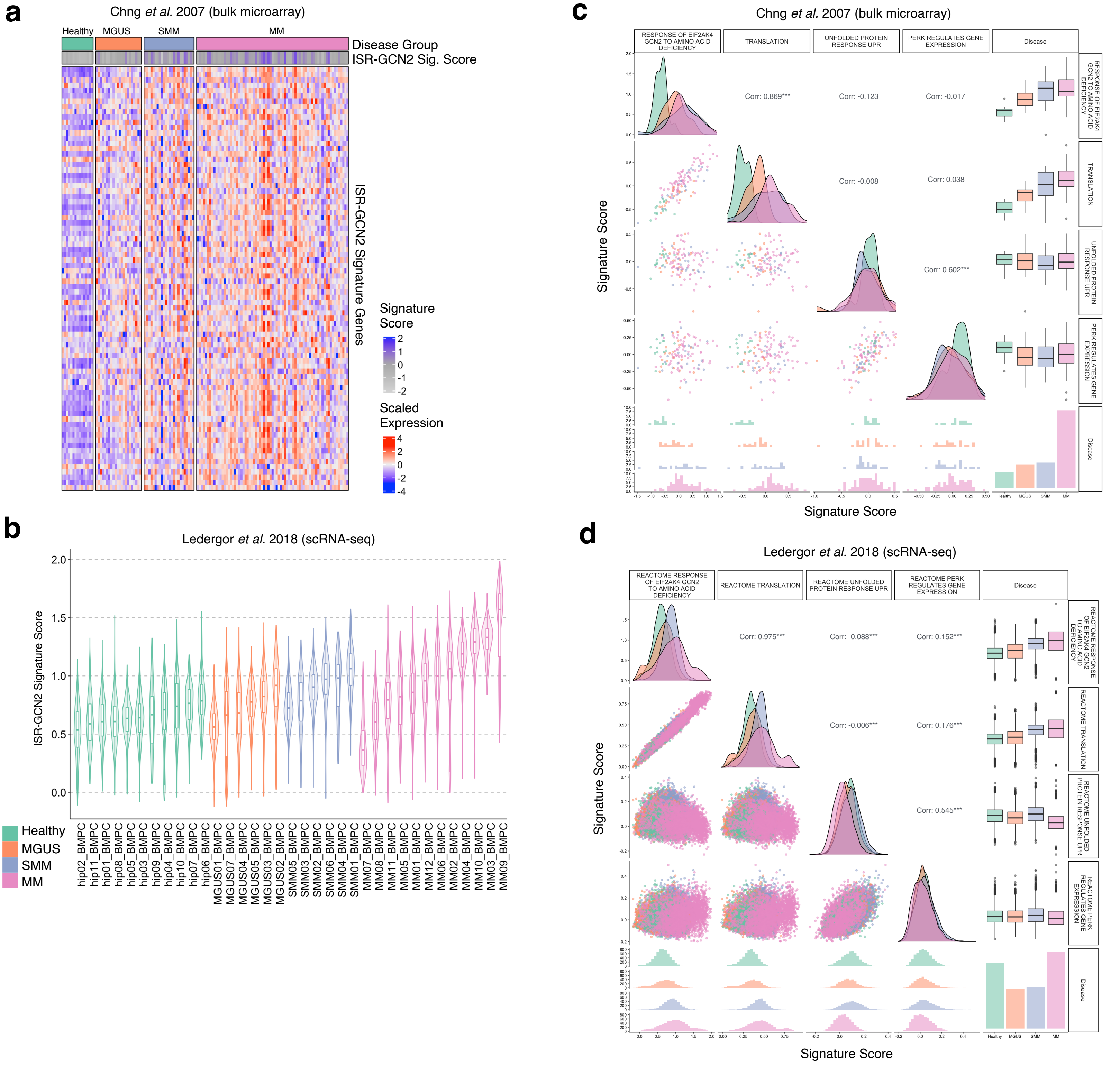

Supplementary Figure 7: Role for ISR-GCN2 pathway activation in myeloma patient progression. (a) Heatmap of genes from ISR-GCN2 signature in samples from Chng *et al.* **(b)** Gene signature scoring for ISR-GCN2 gene set calculated using Seurat's AddModuleScore in Ledergor *et al.* across individual patients. **(c)** Pairwise-correlations between listed gene signature scores computed for each sample from Chng *et al*. using mean of scaled expression values for genes from the indicated gene set. **(d)** Pairwise-correlations between listed gene signature scores computed for each cell from Ledergor *et al.* using Seurat's AddModuleScore. The corresponding Pearson correlations for (c) and (d) are listed with significance (***P<0.001). Boxplots in (b), (c), and (d) represent the distribution of each measurement within defined groups, where the central rectangle spans the interquartile range, the central line represents the median, and "whiskers" above and below the box show the value 1.5x the interquartile range. Source data are provided in SourceData_FigS7.xlsx.

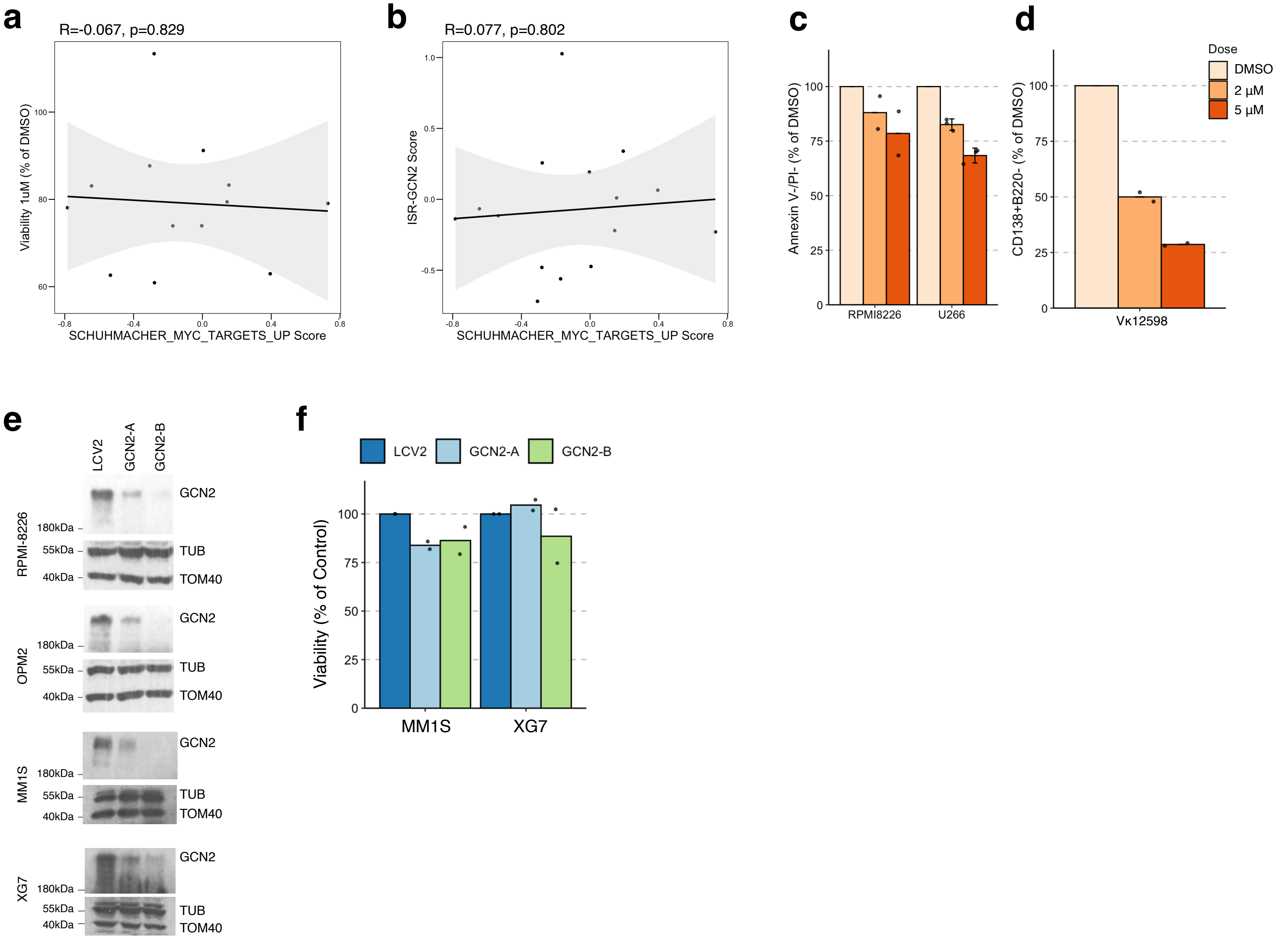

Supplementary Figure 8: *In vitro* analysis of GCN2 blockade in myeloma. (a) Pearson correlation (cor.test, two-sided) between MYC transcriptional activity (determined using Schuhmacher *et al*. gene signature, x-axis) and viability relative to DMSO (1 µM GCN2iB, y-axis). **(b)** Pearson correlation (cor.test, two-sided) between MYC transcriptional activity (determined using Schuhmacher *et al*. gene signature, x-axis) and the ISR-GCN2 gene signature score (y-axis). For both (a) and (b), the linear regression line is plotted in black with confidence interval shaded grey (each dot represents one HMCL). **(c)** Flow cytometric analysis of apoptosis in RPMI8226 and U266 cells after treatment with GCN2iB. **(d)** Flow cytometric analysis of apoptosis in Vκ12598 tumour cells ($CD138^+/B220^-$) after treatment with GCN2iB. Samples in (c) and (d) were collected after 48 hours of treatment with GCN2iB and data represent mean of biological replicates (n=2 for RPMI8226 and Vκ12598, n=3 for U266) with error bars representing standard deviation shown for samples with more than 2 biological replicates. **(e)** Western blot analysis of GCN2 knockout HMCLs. Shown is a representative replicate from two independent experiments. **(f)** Bar plots depicting cell survival, as determined by trypan blue assay, in GCN2 knockout HMCLs that demonstrated insensitivity to GCN2iB. Bar heights represent mean relative viability from two independent experiments (as shown by individual data points). Source data are provided in SourceData_FigS8.xlsx.
