# Supplementary material for: Longitudinal single-cell analysis of a myeloma mouse model identifies subclonal molecular programs associated with progression
Source: Nat Commun. 2021 Nov 3;12:6322. doi: 10.1038/s41467-021-26598-w (PMC8566524; doi:10.1038/s41467-021-26598-w)
Supplement: Supplementary file 4 — Reporting Summary [file 41467_2021_26598_MOESM4_ESM.pdf]

nature research

Corresponding author(s): Trevor J. Pugh and Suzanne Trudel

Last updated by author(s): Sep 10, 2021

Reporting Summary

Nature Research wishes to improve the reproducibility of the work that we publish. This form provides structure for consistency and transparency in reporting. For further information on Nature Research policies, see our Editorial Policies and the Editorial Policy Checklist.

Statistics

For all statistical analyses, confirm that the following items are present in the figure legend, table legend, main text, or Methods section.

n/a	Confirmed	
☐	☒	The exact sample size (n) for each experimental group/condition, given as a discrete number and unit of measurement
☐	☒	A statement on whether measurements were taken from distinct samples or whether the same sample was measured repeatedly
☐	☒	The statistical test(s) used AND whether they are one- or two-sided<br>*Only common tests should be described solely by name; describe more complex techniques in the Methods section.*
☐	☒	A description of all covariates tested
☐	☒	A description of any assumptions or corrections, such as tests of normality and adjustment for multiple comparisons
☐	☒	A full description of the statistical parameters including central tendency (e.g. means) or other basic estimates (e.g. regression coefficient) AND variation (e.g. standard deviation) or associated estimates of uncertainty (e.g. confidence intervals)
☐	☒	For null hypothesis testing, the test statistic (e.g. F, t, r) with confidence intervals, effect sizes, degrees of freedom and P value noted<br>*Give P values as exact values whenever suitable.*
☒	☐	For Bayesian analysis, information on the choice of priors and Markov chain Monte Carlo settings
☒	☐	For hierarchical and complex designs, identification of the appropriate level for tests and full reporting of outcomes
☐	☒	Estimates of effect sizes (e.g. Cohen's d, Pearson's r), indicating how they were calculated

Our web collection on statistics for biologists contains articles on many of the points above.

Software and code

Policy information about availability of computer code

Data collection	No software was used to collect VkMYC single-cell RNA-sequencing data (see below).<br><br>Flow cytometric data was collected using FACSDiva v8.0.1.
Data analysis	The following software and packages were used for processing and analysis of single cell RNA-sequencing data: CellRanger v2, Bamtaghistogram v1-2.12, dropbead v0.3.1, Seurat v3.2.1 and v3.2.2, R v3.6.1, DoubletFinder v2.0.3, KneeArrower v.0.1.0, harmony v1.0, SingleR v1.0.6, inferCNV v1.2.1, ComplexHeatmap v2.2.0<br><br>Code supporting this study is available at https://github.com/pughlab/scVkMYC_mPC.<br><br>Analysis of flow cytometric data was performed using FlowJo v10.7.1.<br><br>Quantification of serum protein electrophoresis for M-protein estimation was performed using ImageJ v1.52a<br><br>Plotting and statistical analyses were performed in the R statistical environment (v3.6.1).

For manuscripts utilizing custom algorithms or software that are central to the research but not yet described in published literature, software must be made available to editors and reviewers. We strongly encourage code deposition in a community repository (e.g. GitHub). See the Nature Research guidelines for submitting code & software for further information.

Data

Policy information about availability of data

All manuscripts must include a data availability statement. This statement should provide the following information, where applicable:

- Accession codes, unique identifiers, or web links for publicly available datasets
- A list of figures that have associated raw data
- A description of any restrictions on data availability

"The scRNA-seq data generated in this study have been deposited as raw bam files and as processed gene expression matrices at the National Center for Biotechnology Information Gene Expression Omnibus with accession numbers SRP214856 and GSE134370 [https://www.ncbi.nlm.nih.gov/geo/query/acc.cgi?acc=GSE134370]. Source data for all figures are also provided for this paper."

Previously published single-cell and bulk RNA-sequencing data from patients that were re-analyzed in this study were acquired via NCBI Gene Expression Omnibus including Chng et al. 2007 (GSE6477) and Ledergor et a. 2018 (GSE117156).

Bulk RNA-seq data for human myeloma cell lines were obtained from the Keats Lab repository using https://www.keatslab.org/data-repository (HMCL66_Gene_Expression_FPKM).

Field-specific reporting

Please select the one below that is the best fit for your research. If you are not sure, read the appropriate sections before making your selection.

☒ Life sciences ☐ Behavioural & social sciences ☐ Ecological, evolutionary & environmental sciences

For a reference copy of the document with all sections, see nature.com/documents/nr-reporting-summary-flat.pdf

Life sciences study design

All studies must disclose on these points even when the disclosure is negative.

Sample size	Sample size was determined by the availability of subjects for the single-cell RNA-sequencing portion of this study, but a minimum of 3 samples for each disease stage group was decided upon upfront to capture biological heterogeneity between tumours.
Data exclusions	All single-cell RNA-sequencing data acquired for this study was pre-processed and utilized to identify plasma cells in the data. Since 2 early-MM samples contained too few malignant cells, they were excluded from downstream analysis. For all samples, doublets were excluded and low-quality cells were removed if they contained <500 genes, <1000 transcripts, and/or >15% mitochondrial transcripts. Exclusion criteria was not pre-established.
Replication	Technical replicates were not performed for single cell RNA-sequencing experiments as samples were limited. For in vitro cell line experiments, 2-4 independent replicates were performed. For CRISPR knockout experiments, two independent constructs were used and viability measurements were obtained in duplicate. All attempts at replication were successful for in vitro and CRISPR knockout experiments.
Randomization	The study design was observational only and did not involve allocating subjects into treatment groups. Thus, randomization was not relevant to the study design.
Blinding	The study design was observational only and did not involve allocating subjects into treatment groups. Thus, blinding was not relevant to the study design.

Reporting for specific materials, systems and methods

We require information from authors about some types of materials, experimental systems and methods used in many studies. Here, indicate whether each material, system or method listed is relevant to your study. If you are not sure if a list item applies to your research, read the appropriate section before selecting a response.

Materials & experimental systems

n/a	Involved in the study	
☐	☒	Antibodies
☐	☒	Eukaryotic cell lines
☒	☐	Palaeontology and archaeology
☐	☒	Animals and other organisms
☒	☐	Human research participants
☒	☐	Clinical data
☒	☐	Dual use research of concern

Methods

n/a	Involved in the study	
☒	☐	ChIP-seq
☐	☒	Flow cytometry
☒	☐	MRI-based neuroimaging

Antibodies

Antibodies used	CD138-APC: BD Biosciences, cat.558626, clone.281-2<br>B220-PE: BD Biosciences, cat.553089, clone.RA3-6B2<br>FITC Annexin V Apoptosis Detection Kit: BD Biosciences, cat.556547<br>GCN2 (1:1000): Cell Signaling Technologies, cat.3302<br>TOM40 (1:1000): Proteintech, cat.18409<br>Beta-Tubulin (1:1000): Cell Signaling Technologies, cat.2146<br>HRP-conjugated anti-Rabbit (1:1000): Cytiva, cat.NA934
Validation	Antibodies are validated by manufactures.<br><br>Flow antibodies from BD Biosciences are pre-titrated and tested by flow cytometric analysis of C57BL/6 bone-marrow leukocytes (CD138-APC), mouse splenic leukocytes (B220-PE), or Jurkat T cells treated with or without campotothecin to induce apoptosis (Annexin V-FITC). GCN2 antibody from Cell Signaling Technologies is validated by western blot analysis of extracts from ME180 and HT1376 cells that were untreated, treated with UV light (50mJ/cm2, 30 minutes), or subjected to nocodazole block (50 ng/ml, 24hrs), using GCN2 Antibody. B-Tubulin antibody from Cell Signaling Technologies is validated by western blot analysis of extracts from various cell lines. TOM40 antibody from Proteintech is validated by western blot analysis of extracts from HEK-293, HeLa, and HepG2.

Eukaryotic cell lines

Policy information about cell lines

Cell line source(s)	Human myeloma cell lines were obtained from ATCC (MM1S, RPMI-8226, U266), DMSZ (AMO1, JJN3, OPM2), and the Ontario Cancer Institute (OCI-MY5).<br><br>XG6 and XG7 were provided by Prof. L. Bergsagel (Mayo Clinic, Scottsdale, AZ)<br><br>HEK293T were obtained from ATCC.
Authentication	All cell lines were authenticated by PCR-based STR fingerprinting.
Mycoplasma contamination	All cell lines were tested and confirmed negative for mycoplasma contamination.
Commonly misidentified lines<br>(See ICLAC register)	No commonly misidentified cell lines were used in this study.

Animals and other organisms

Policy information about studies involving animals; ARRIVE guidelines recommended for reporting animal research

Laboratory animals	The cohort of mice used in this study was comprised of 15 Vκ*MYC mice (aged 27-74 weeks) cross-bred onto C57BL/KaLwRij and 3 C57BL/KaLwRij control mice (aged 55-72 weeks). The study used both male and female mice (refer to Supplementary Data 1 for details).
Wild animals	The study did not involve wild animals.
Field-collected samples	The study did not involve samples collected from the field.
Ethics oversight	Animals used in this study were housed in pathogen-free facilities at either the Montreal University Health Centre (MUHC) or University Health Network (UHN). All related experiments were approved by institutional Animal Care Committees and performed in accordance with the Canadian Council on Animal Care Guidelines (UHN AUP#958.23, MUHC AUP#2012-7242).

Note that full information on the approval of the study protocol must also be provided in the manuscript.

Flow Cytometry

Plots

Confirm that:

☒ The axis labels state the marker and fluorochrome used (e.g. CD4-FITC).

☒ The axis scales are clearly visible. Include numbers along axes only for bottom left plot of group (a 'group' is an analysis of identical markers).

☒ All plots are contour plots with outliers or pseudocolor plots.

☒ A numerical value for number of cells or percentage (with statistics) is provided.

Methodology

Sample preparation	Human myeloma cell lines and Vκ12598 cells were collected as a single cell suspension and stained with antibodies for 30

Sample preparation	minutes unless otherwise stated by manufacturer
Instrument	BD FACS Canto II
Software	Data were collected using FACSDiva v8.0.1 and analyzed using FlowJo v.10.7.1.
Cell population abundance	Flow cytometry experiments were analytical only and did not sort on any cell populations.
Gating strategy	Human myeloma cell lines were gated to identify Annexin V-/PI- quadrant. Vκ12598 myeloma cells were gated as CD138+/B220-.

☒ Tick this box to confirm that a figure exemplifying the gating strategy is provided in the Supplementary Information.
